# Fractionation and Functional Characterization of *Limnospira platensis* Extracellular Polysaccharides as Potential Food Ingredients from Recycled Cultivation Media

**DOI:** 10.3390/foods15101801

**Published:** 2026-05-19

**Authors:** Zihan Li, Chen Sang, Yuhuan Liu, Roger Ruan, Qi Zhang

**Affiliations:** 1 State Key Laboratory of Food Science and Technology, Engineering Research Center for Biomass Conversion, Ministry of Education, College of Food Science and Technology, Nanchang University, Nanchang 330047, China; 352313320026@email.ncu.edu.cn (Z.L.); 407900250145@email.ncu.edu.cn (C.S.); zhangqi093115@ncu.edu.cn (Q.Z.); 2 Center for Biorefining, Department of Bioproducts and Biosystems Engineering and Department of Food Science and Nutrition, University of Minnesota, Saint Paul, MN 55108, USA; ruanx001@umn.edu

**Keywords:** *Limnospira platensis*, medium recycling, extracellular organic matter, molecular weight heterogeneity

## Abstract

*Limnospira platensis* is a promising sustainable biomass for functional food production. During cultivation, it secretes extracellular polysaccharides (EPS) with underutilized potential as food ingredients. This study aimed to strategically fractionate *Spirulina* EPS (SEPS) by molecular weight (MW: <30, 30–100, >100 kDa) to elucidate their structure-function relationships for targeted food applications. We found distinct functional diversification: The mid-MW fraction (SEPS-2, 30–100 kDa) was an amphiphilic glycoprotein complex with potential interfacial activity. The high-MW fraction (SEPS-3, >100 kDa) formed a dense, glucose-rich glucan network, suggesting utility as a natural thickener or texturizer. In contrast, the low-MW fraction (SEPS-1, <30 kDa), rich in deoxy-sugars, exhibited superior antioxidant capacity, indicating potential as a bioactive preservative or nutraceutical. Spectroscopic and morphological analyses linked these structural differences to their physicochemical properties. Notably, the 30–100 kDa fraction transitions from a cultivation byproduct to a functional food architect, where its interfacial properties can be leveraged to engineer stable, clean-label emulsion-based food products. This work provides a foundation for the valorization of *L. platensis* EPS, demonstrating how MW-directed fractionation can unlock tailored functionalities-from bioactive agents to structural polymers-for the development of next-generation foods from circular bioeconomy streams.

## 1. Introduction

*Spirulina* offers exceptional nutritional value [1], yet its commercial potential is constrained by production costs of 10–30 USD/kg, driven largely by intensive freshwater and nutrient consumption [2,3]. With roughly one-third of all food produced for human consumption being lost or wasted, the development of novel food ingredients from microalgae by-products has emerged not merely as a niche area of research, but as a defining and indispensable trend in contemporary food science [4,5]. The commercially cultivated “*Spirulina*” was historically classified under *Spirulina* or *Arthrospira*, but is now formally transferred to the new genus *Limnospira* [6]. To ensure taxonomic accuracy and nomenclatural consistency throughout this study, the taxonomically rigorous designation *Limnospira platensis* (*L. platensis*) is adopted uniformly in the present paper.

To align with circular bioeconomy principles, the industry is shifting toward recycling cultivation modes [7], which can reduce freshwater demand by 84% and nutrient requirements by 55% [8]. Recycling harvesting effluent not only minimizes source water withdrawal and energy consumption but also prevents the eutrophication of receiving water bodies caused by the discharge of unutilized nutrients. It has been widely reported that organic matters (OMs) within the recycled media accumulates as the number of recycling cycles increases [9,10,11]. And the regulatory effects of OMs on algal physiology are highly dependent on their molecular weight (MW) distribution [12]. Notably, removing OMs >30 kDa mitigates growth inhibition after five recycling iterations, yet isolating only the >100 kDa fraction fails to fully alleviate this suppression [13]. These findings underscore a critical functional divergence within the 30–100 kDa OMs fraction. Extracellular polysaccharides (EPS) is the primary functional component of OMs [11]. The biological potency of these fractions is closely tied to their MW-dependent molecular mobility and radical accessibility [14]. In food science, MW affects solubility, viscosity, and fermentability, which in turn influence mouthfeel, satiety, and prebiotic potential [15]. Polysaccharides with high MW (>100 kDa) often form secondary structures like triple helices, which enhance their biological activity through specific interactions with immune receptors [16]. Lower MW polysaccharides are typically more fermentable by gut microbiota, impacting gut health and nutrient absorption. As such, accurate MW determination is essential for tailoring polysaccharide functionality across industrial, nutritional, and therapeutic applications [17]. However, the structural difference and functional compartmentalization of different MW EPS fractions recovered from large-scale recycling systems-where polydispersity is significantly more complex-remain largely unexplored.

*L. platensis* polysaccharides have revealed potent antioxidant and α-glucosidase inhibitory activities, particularly in fractions obtained through controlled degradation, indicating EPS do not behave as a homogeneous entity [18,19]. Moreover, *L. platensis*-derived EPS are known for diverse bioactivities, including antioxidant [20], anticoagulant [21], antibacterial [22,23], antiviral [24] and anti-inflammatory properties [25], alongside promising prebiotic potential [26], positioning them as high-value candidates in food engineering, cosmeceuticals, and pharmaceuticals. Instead, there exists an inherent functional compartmentalization within the EPS pool, where biological and physicochemical properties are strictly partitioned according to their MW. However, most existing studies treat these substances as a collective whole during characterization, thereby overlooking the significant chemical differentiation among components with varying degrees of polymerization. This study therefore hypothesizes that the bioactivity of EPS is compartmentalized into three distinct MW windows: <30 kDa, 30–100 kDa, and >100 kDa, with the 30–100 kDa fraction representing an optimal functional window.

To bridge this knowledge gap, the SEPS (*Spirulina* EPS) used in this study were harvested from industrial-scale open raceway ponds (Jiangxi Zhongzao Bio-technology). Unlike controlled laboratory micro-environments, these industrial samples provide a more authentic reflection of the metabolic imprints left by *L. platensis* under complex ecological stressors, such as fluctuating irradiance and hydrodynamic shear [27,28]. By systematically characterizing the structural divergence across different molecular weight (MW) intervals, this research establishes a critical physiochemical foundation for SEPS-based ingredients. To this end, a systematic “fractionation-elucidation-evaluation” strategy was implemented, using membrane ultrafiltration to isolate three distinct fractions: SEPS-1 (<30 kDa), SEPS-2 (30–100 kDa), and SEPS-3 (>100 kDa). Through a multi-dimensional characterization framework, this study aims to decode the structure-activity relationships within the SEPS pool. By unveiling the intrinsic nexus between MW distribution and biochemical functionality, this study provides a fundamental basis for tailoring specific fractions to distinct food applications. Ultimately, this work facilitates the transition from cultivation by-products to high-value functional ingredients, supporting precision management in sustainable microalgal biorefineries.

## 2. Materials and Methods

### 2.1. Materials

The experimental samples were obtained from the large-scale *L. platensis* cultivation base of Jiangxi Zhongzao Bio-technology Co., Ltd. (Ruijin City, Jiangxi Province, China). Following the biomass harvesting process at the exponential growth phase, large volumes of the cultivation effluent were collected in situ. The collected culture media were initially subjected to vacuum filtration using 0.45 μm Whatman filter membranes to thoroughly remove residual algal cells and fine suspended solids, yielding cell-free culture supernatants. Subsequently, the harvesting recycling medium was processed for the extraction of EPS following the protocol described by Li et al. [12].

### 2.2. EPS Fractions Ultrafiltration

Extracted EPS was reconstituted in ultrapure water and further fractionated via sequential ultrafiltration to characterize its heterogeneous molecular weight distribution. Three distinct *L. platensis* EPS (SEPS) fractions were systematically isolated: SEPS-1 (<30 kDa), SEPS-2 (30–100 kDa), and SEPS-3 (>100 kDa). Following the freezing and lyophilization process, the resulting SEPS series featured well-defined molecular weight gradients.

### 2.3. Chemical Characterization of SEPS

#### 2.3.1. Element Analysis

Elemental abundances of the SEPS fractions were determined using an elemental analyzer (Vario EL III, Elementar, Langenselbold, Germany). To evaluate their structural characteristics, the elemental composition was further characterized by calculating the mass fraction ratios of C, H, N, and S.

#### 2.3.2. Evaluation of Physicochemical Properties of SEPS

The total carbohydrate content of the SEPS fractions was determined using the phenol-sulfuric acid method, with D-glucose as the external standard [29]. The uronic acid content was quantified via the carbazole-sulfuric acid method at 525 nm, employing D-galacturonic acid as the standard [30]. Additionally, the protein concentration was analyzed using the Bradford assay (Coomassie Brilliant Blue G-250 binding method), with bovine serum albumin (BSA) utilized to construct the standard curve [31].

#### 2.3.3. Determination of Monosaccharide Composition

Monosaccharide compositions of the SEPS fractions were determined using a Dionex ICS-5000 High-Performance Anion-Exchange Chromatography system (Thermo Fisher Scientific, Waltham, MA, USA). Briefly, 5 mg of each sample was subjected to pre-hydrolysis with 12 M H_2_SO_4_ in an ice bath for 0.5 h, followed by complete hydrolysis at 100 °C for 2 h after dilution to a final acid concentration with 2.0 mL of ultrapure water. The resulting hydrolysate was neutralized and filtered through a 0.45 μm membrane prior to injection. Chromatographic separation was achieved using a CarboPac™ (Thermo Fisher Scientific, Sunnyvale, CA, USA) PA20 analytical column (3 × 150 mm) equipped with a guard column (3 × 30 mm). Gradient elution was performed at a flow rate of 0.5 mL/min (30 °C) using a mobile phase consisting of 0.25 M NaOH, ultrapure water, and 1.0 M NaHCO_3_. Monosaccharides were detected via Pulsed Amperometric Detection with a gold working electrode. Each 10 μL injection had a total run time of 56 min, and data processing was conducted using Chromeleon 6.80 software.

#### 2.3.4. Ultraviolet-Visible (UV) and Fourier-Transform Infrared (FT-IR) Analysis

For UV-Vis spectroscopic analysis, a 2 mg/mL aqueous solution of the SEPS fractions were prepared with deionized water and scanned using a UV-Vis spectrophotometer (UV9100, Shanghai Metash Instruments Co., Ltd., Shanghai, China) across a wavelength range of 190–800 nm. For FT-IR characterization, the lyophilized SEPS fractions were blended with KBr at a weight ratio of 1:100 and pressed into translucent pellets. Spectra were recorded using an FT-IR spectrometer (Nicolet iS5, Thermo Scientific, Madison, WI, USA) in the wavenumber range of 400–4000 cm^−1^ at a resolution of 4 cm^−1^ with 64 co-added scans.

#### 2.3.5. X-Ray Photoelectron Spectroscopy (XPS)

Surface elemental compositions and the chemical environments of functional groups within the SEPS fractions were characterized via X-ray Photoelectron Spectroscopy (XPS). Lyophilized SEPS fractions were vacuum-dried to eliminate adventitious water, uniformly immobilized onto conductive carbon tape, and subsequently loaded into a pre-vacuum chamber. During analysis, a dual-beam charge neutralizer was activated for effective charge compensation. Spectra were acquired at pass energies of 20 eV for high-resolution scans and 40 eV for survey scans, with the binding energy scale calibrated to the adventitious C 1s peak at 284.8 eV. Data processing was conducted using Avantage software (version 5.9921). The spectral background was subtracted using the ‘Smart’ algorithm, and narrow-scan spectra were deconvoluted using a Gaussian-Lorentzian sum function (30% Lorentzian character) to ensure precise peak assignment.

#### 2.3.6. Scanning Electron Microscopy (SEM) Analysis

Lyophilized SEPS fractions were uniformly mounted onto aluminum stubs using double-sided conductive carbon tape and subsequently sputter-coated with a thin layer of gold in a vacuum evaporator to enhance surface conductivity. Morphological observations were conducted using a Field-Emission scanning electron microscopy (SEM) system (Regulus 8100, Hitachi, Tokyo, Japan) at an accelerating voltage of 5 kV. High-resolution micrographs were systematically captured at magnifications of 5000× and 20,000× to analyze both the overall topography and fine structural details of the SEPS.

#### 2.3.7. X-Ray Diffraction (XRD)

X-ray diffraction patterns of SEPS fractions were recorded using a D8 ADVANCE diffractometer (Bruker, Ettlingen, Germany) equipped with a Cu Kα radiation source. The analysis was performed at an accelerating voltage of 40 kV and an applied current of 30 mA. Each sample was scanned across a 2θ range of 3–90° at a constant scan rate of 2°/min. The relative crystallinity (X_c_) was subsequently quantified using JADE 6.0 software, defined as the ratio of the crystalline peak area to the total integrated diffraction area.

### 2.4. Congo Red Experiment

The triple-helical conformation of the SEPS fractions was evaluated using the Congo red assay [32]. Briefly, 100 μL of each SEPS solution (1 mg/mL in deionized water) was mixed with an equal volume of 80 μM Congo red. The resulting mixture was then incorporated into NaOH solutions to achieve a series of final concentrations ranging from 0 to 2.0 M. The maximum absorption wavelength (λ_max_) was monitored using a microplate reader. A characteristic red-shift or blue-shift in λ_max_ as a function of NaOH concentration was used to identify the transition and stability of the triple-helical structure.

### 2.5. Thermal Stability

Thermal degradation behaviors of the SEPS fractions were investigated using a DTG-60H thermogravimetric analyzer (Shimadzu, Kyoto, Japan) to evaluate their thermal stability and compositional characteristics. Approximately 5 mg of each lyophilized sample was accurately weighed into an alumina crucible and heated from ambient temperature to 950 °C at a constant heating rate of 10 °C/min under a continuous nitrogen atmosphere. The residual mass (TG curve) was recorded synchronously, while the derivative thermogravimetric (DTG) curve was obtained via first-order differentiation of the TG data to characterize the maximum mass loss rate.

### 2.6. Rheological Characterization

Rheological properties of the SEPS fractions (2.5%, *w*/*v*) were characterized using an Anton Paar MCR 302 rheometer with a PP25 parallel plate (25 mm diameter, 1.0 mm gap) at 25 °C. To evaluate the viscoelastic limits, amplitude sweeps were performed at a fixed frequency of 1 Hz, with the shear strain (γ) increasing from 0.001 to 10 (10^−3^ to 10, expressed as log_10_γ). The storage modulus (G′), loss modulus (G″), and loss tangent (tanδ) were recorded to determine the linear viscoelastic (LVE) region. Subsequently, frequency sweeps were conducted from 0.1 to 100 rad/s (expressed as 10^−1^ to 10^2^ in log_10_scale) at a constant strain of 1%. Furthermore, steady shear tests were executed in controlled shear rate (CSR) mode, with the shear rate (γ̇) varying from 0.1 to 100 s^−1^ (10^−1^ to 10^2^ in log_10_ scale). The apparent viscosity (η, mPa·s) and shear stress (τ, Pa) were monitored to assess the flow behavior and pseudoplasticity of the SEPS fractions.

### 2.7. Antioxidant Activities Assays

The radical scavenging capacities of SEPS fractions were evaluated against ABTS and DPPH radicals, with L-ascorbic acid (V_C_) serving as the positive control. All measurements were performed in triplicate, and results were expressed as scavenging rates (%).

#### 2.7.1. Scavenging Capacity of ABTS Radical

The ABTS scavenging capacity was determined according to the protocol of Ktari et al. [33] with modifications. The ABTS^+^·working solution was generated by reacting 7.0 mM ABTS with 2.45 mM potassium persulfate (1:1, *v*/*v*) for 12–16 h in the dark. Before use, the solution was diluted with 0.1 M phosphate-buffered saline (PBS, pH 7.4) to an absorbance of 0.70 ± 0.02 at 734 nm. Briefly, a 0.2 mL aliquot of the sample at varying concentrations (0.05, 0.10, 0.25, 0.50, 1.00, 2.50 mg/mL) was mixed with 3.8 mL of the ABTS^+^·working solution. After vigorous vortexing and a 6-min incubation at room temperature in the dark, the absorbance was recorded at 734 nm (*A_sample_*). The blank control (*A_control_*) utilized deionized water instead of the sample, while the sample background (*A_background_*) replaced the ABTS^+^·working solution with an equal volume of 0.1 M PBS.

#### 2.7.2. Scavenging Capacity of DPPH Radical

The DPPH scavenging activity was determined according to the method described by Nyaisaba et al. [34] with minor modifications. A 0.1 mmol/L DPPH working solution was prepared in anhydrous ethanol and stored in the dark. SEPS samples were dissolved in deionized water to achieve a concentration gradient (0.05, 0.10, 0.25, 0.50, 1.00, 2.50 mg/mL). Briefly, 2.0 mL of the sample solution was mixed with 2.0 mL of the DPPH working solution. Following vigorous vortexing, the mixture was incubated in the dark at room temperature for 30 min. The absorbance was measured at 517 nm (*A_sample_*). Two control groups were established: a blank control (*A_control_*) consisting of 2.0 mL DPPH solution and 2.0 mL deionized water, and a sample background (*A_background_*) comprising 2.0 mL sample solution and 2.0 mL anhydrous ethanol.

The radical scavenging rates (SR, %) for both DPPH and ABTS assays were calculated according to the following equation:

(1) $$SR(\%)=\left(1-\frac{A_{sample}-A_{backrgound}}{A_{control}}\right)\times 100\%$$

### 2.8. Data Processing and Statistical Analysis

Data were analyzed using SPSS 26.0, with mean comparisons evaluated via Tukey’s post-hoc test at a significance level of *p* < 0.05. All experiments were performed in triplicate and results are expressed as mean ± SD. Graphical illustrations were generated using Origin Pro 2022, where distinct letters denote statistically significant differences between groups.

## 3. Results

### 3.1. Elemental Composition

The elemental compositions and stoichiometric ratios of the SEPS fractions are summarized in Table 1. Distinct elemental distributions were observed among the different molecular weight fractions (*p* < 0.05). As shown in Table 1, the C/N ratios ranged from 9.207 to 10.015, with SEPS-3 exhibiting the highest value (10.015 ± 0.009). This elevated C/N ratio in the high-molecular-weight fraction suggests a reduction in nitrogen-containing proteinaceous impurities, thereby indicating higher polysaccharide purity. Conversely, SEPS-1 possessed the lowest C/N ratio (9.207 ± 0.614), reflecting a relatively higher proportion of protein-associated moieties. Regarding the hydrogen distribution, SEPS-1 displayed the highest C/H ratio (5.738 ± 0.134), significantly exceeding those of SEPS-2 and SEPS-3, which may imply a higher degree of structural complexity or unsaturation in the low-molecular-weight fraction. Consistently, the H/N ratios of SEPS-2 (1.878 ± 0.068) and SEPS-3 (1.945 ± 0.043) were notably higher than that of SEPS-1 (1.607 ± 0.145). Of particular note, the sulfur (S) content across all fractions remained below 0.3%, characterizing these SEPS as non-sulfated or ultra-low sulfur polymers. These elemental variations within the SEPS fractions directly reflect significant heterogeneities in polysaccharide purity and associated protein content across different molecular weight distributions.

### 3.2. Biochemical Composition and Monosaccharide Profiling

**Figure 1:**
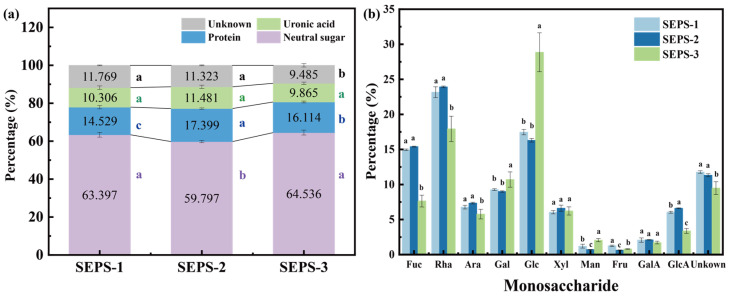
Biochemical Composition (**a**) and Monosaccharide Profiling (**b**) of SEPS fractions. Different lowercase letters (a–c) above the bars indicate significant differences among the three fractions within the same category (*p* < 0.05). Error bars represent standard deviation (SD) from three independent replicates.

The integration of qualitative biochemical analysis (Figure 1a) and high-resolution monosaccharide profiling (Figure 1b) revealed pronounced structural heterogeneity and functional differentiation among the SEPS fractions. Biochemical assays indicated that SEPS-2 (30–100 kDa) was characterized by a significantly higher protein content (17.40%, *p* < 0.05) and the lowest neutral sugar proportion (59.80%) among all fractions. In contrast, SEPS-3 (>100 kDa) exhibited a shift toward a more refined glycan backbone, with neutral sugar content significantly increasing to 64.54% (*p* < 0.05). SEPS-1 (<30 kDa) represented a transitional state, featuring a neutral sugar content (63.40%) comparable to SEPS-3 but with the lowest recorded protein levels (14.53%). Monosaccharide composition analysis (Figure 1b) identified SEPS as a complex acidic heteropolysaccharide composed of ten distinct constituents: fucose (Fuc), rhamnose (Rha), glucose (Glc), galactose (Gal), mannose (Man), fructose (Fru), arabinose (Ara), xylose (Xyl), glucuronic acid (GlcA), and galacturonic acid (GalA). The observed monosaccharide profile is consistent with previously reported compositions of exopolysaccharides derived from *L. platensis* [35,36,37,38]. Significant variations in molar ratio were observed between fractions. SEPS-1 and SEPS-2 were enriched in deoxy-sugars, with the combined proportion of Rha and Fuc of 38.12%. Conversely, the structural divergence of SEPS-3 was primarily marked by its glucose content, which increased from 16.30–17.47% in the lower-MW fractions to 28.86% in SEPS-3. Additionally, the proportions of Gal and Man in SEPS-3 rose significantly to 12.58% and a 2.81-fold increase over SEPS-2, respectively, while the total uronic acid (GalA + GlcA) content remained statistically similar across all fractions.

### 3.3. FT-IR Spectroscopic and UV-VIS Spectroscopic Analysis

**Figure 2:**
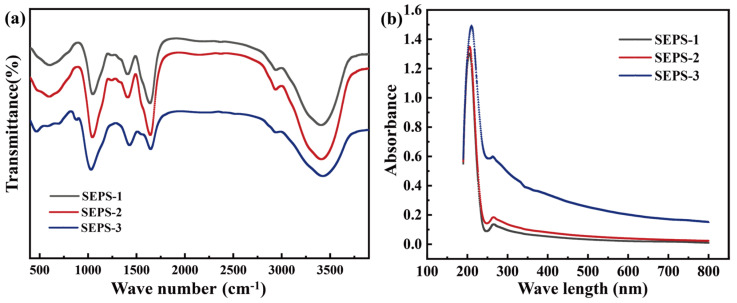
FT-IR spectra (**a**) and UV-VIS spectra (**b**) of SEPS fractions.

All three SEPS fractions exhibited characteristic polysaccharide absorption features within the 4000–400 cm^−1^ range (Figure 2a), although significant structural heterogeneities were observed in peak intensities and profiles. A broad, intense absorption band near 3450 cm^−1^ was attributed to the stretching vibrations of intra- and intermolecular hydroxyl groups (-OH), with a low-frequency shoulder at approximately 3240–3238 cm^−1^ corresponding to the amine (-NH) stretching vibrations of protein moieties [39]. SEPS-2 displayed the highest absorption intensity in this region, consistent with its elevated protein content. In the 2950 cm^−1^ region, C-H stretching vibrations followed the intensity trend of SEPS-1 ≈ SEPS-2 > SEPS-3. Prominent peaks at 1652 cm^−1^ and 1416 cm^−1^ were observed in all fractions, corresponding to the asymmetric and symmetric stretching vibrations of carboxylate groups (-COO^-^), with SEPS-2 displaying the most pronounced response. In the polysaccharide fingerprint region (1200–950 cm^−1^), SEPS-2 featured a sharp, intense peak at 1046 cm^−1^ (C-O-C glycosidic bond vibrations), while SEPS-3 exhibited a broader profile and a discernible wavenumber shift, suggesting variations in glycosidic linkages. Absorption bands near 918 cm^−1^ and 820 cm^−1^ are indicative of specific glycosidic linkages, thereby confirming the complex heteropolymeric nature of the SEPS fractions and their mixed-linkage backbone [25]. This spectral profile is highly consistent with the exopolysaccharides (EPS) previously purified from *L. platensis* (formerly *A. platensis*) [25,40].

The UV-visible absorption spectra (Figure 2b) further characterized the electronic transition behaviors of the chromophores. All three fractions exhibited intense absorption peaks in the 200–210 nm region, arising from the n→σ* or π→π* transitions of glycosidic bonds, carboxyl, and hydroxyl groups [35]. Notably, the absorption intensity of SEPS-3 was significantly higher than that of SEPS-1 and SEPS-2 in this far-UV region. In the 260–280 nm range, no sharp isolated peaks were observed; instead, all fractions displayed broad shoulders characteristic of proteoglycan complexes, with SEPS-3 consistently maintaining higher absorbance. Furthermore, in the visible region (300–800 nm), the baseline of SEPS-3 was markedly elevated and showed a gradual attenuation with increasing wavelength. This profile is indicative of significant light scattering, likely stemming from the higher molecular weight and increased aggregation tendency of the SEPS-3 chains.

### 3.4. Thermal Stability Analysis

**Figure 3:**
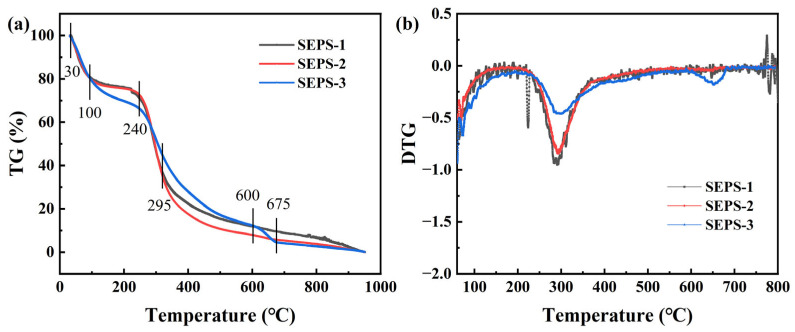
Thermal stability of SEPS fractions: (**a**) TG curves, (**b**) DTG curves.

The thermal stability and degradation kinetics of the SEPS fractions were characterized using thermogravimetric analysis (TGA) and derivative thermogravimetry (DTG), as illustrated in Figure 3.

All three fractions exhibited a characteristic three-stage thermal degradation profile: (I) a dehydration stage (30–200 °C) involving the evaporation of adsorbed and bound water; (II) a primary degradation stage (200–400 °C) corresponding to the depolymerization of glycan chains and cleavage of glycosidic bonds; and (III) a carbonization stage (>400 °C) characterized by carbon skeleton rearrangement and the formation of inorganic residues. Significant variations in the maximum degradation temperatures (T_d,max_) were observed among the fractions. SEPS-1 reached its maximum degradation rate at approximately 255 °C, the lowest among the three groups. SEPS-2 exhibited a higher T_d,max_ of approximately 290 °C, characterized by a sharp, concentrated DTG peak. Notably, despite possessing the significantly lowest ash content (*p* < 0.05), SEPS-3 demonstrated the highest thermal stability and the greatest final residual mass (TG%) at elevated temperatures. Furthermore, SEPS-2 showed a higher cumulative weight loss (approx. 80%) at 400 °C compared to SEPS-1 (approx. 75%), while SEPS-1 yielded the lowest final residual percentage upon completion of the heating program.

### 3.5. XRD Analysis

**Figure 4:**
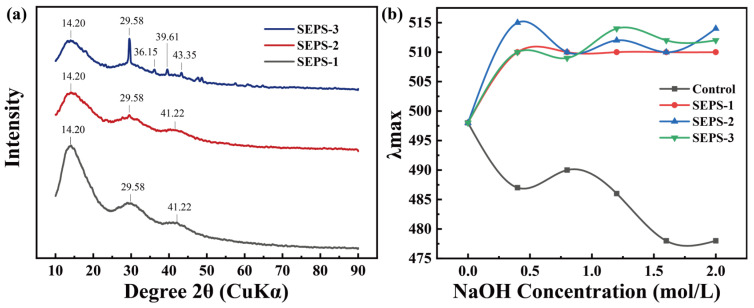
Structural characterization of SEPS fractions: (**a**) X-ray diffraction (XRD) patterns; (**b**) Triple-helix conformation analysis via Congo red test at varying NaOH concentrations.

The micro-spatial arrangement and assembly patterns of the SEPS fractions were further elucidated through X-ray diffraction (XRD) analysis (Figure 4a). All three fractions exhibited characteristic polysaccharide diffraction features, marked by broad, diffuse halos centered near 2θ ≈ 15° and 30°, indicating a coexistence of amorphous matrices and semi-crystalline microdomains. Quantitative analysis performed using Jade software (Table 2) revealed relative crystallinity values ranging from 68.82% to 70.58%. Specifically, SEPS-2 exhibited the highest relative crystallinity (70.58%), followed by SEPS-3 (69.96%), while SEPS-1 showed the lowest degree of crystalline order. Notably, SEPS-3 displayed a relatively sharper diffraction peak near 30° compared to the other fractions, suggesting a higher degree of long-range structural regularity in its high-molecular-weight chains. These variations in diffraction profiles reflect a significant divergence in the self-assembly logic and crystalline evolution among the molecular weight-fractionated SEPS.

### 3.6. Congo Red Assay

The conformational transition of the SEPS fractions were characterized using the Congo red assay (Figure 4b). As the NaOH concentration increased from 0 to 2.0 M, the maximum absorption wavelengths (λ_max_) of the SEPS-Congo Red complexes for all three fractions exhibited a significant red shift, increasing from 498 nm to a range of 509–515 nm. In contrast, the control group showed a characteristic blue-shift under identical conditions. Among the fractions, SEPS-2 demonstrated the highest λ_max_ (515 nm) and the most pronounced red-shift magnitude at 0.4 M NaOH. Notably, SEPS-3 exhibited the superior alkaline stability in the strong base region (1.0–2.0 mol/L), maintaining a λ_max_ of 512 nm even at 2.0 mol/L NaOH, with the most gradual declining trend. Conversely, SEPS-1 maintained the lowest λ_max_ values across the entire concentration range, indicating the weakest conformational stability and a higher susceptibility to alkaline denaturation.

### 3.7. SEM Morphological Analysis

**Figure 5:**
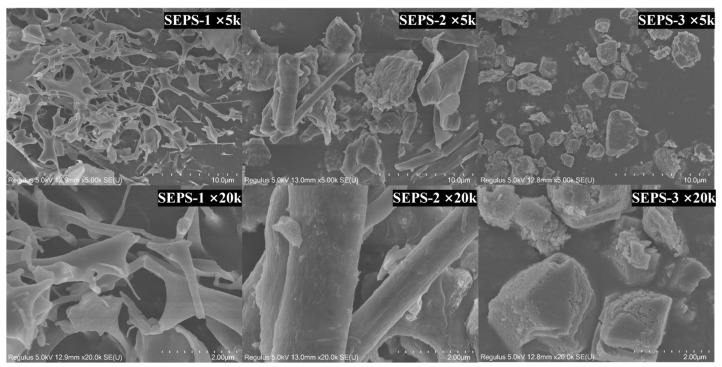
Scanning electron microscopy (SEM) analysis of SEPS fractions at different magnifications. The morphology of SEPS-1, SEPS-2, and SEPS-3 was observed at 5000 times (top row, scale bar = 10.0 μm and 20,000 times (bottom row, scale bar = 2.00 μm).

The micro-surface topographies and spatial arrangements of the SEPS fractions were visualized using SEM (Figure 5). At magnifications of 5000× and 20,000×, SEPS-1 exhibited a loose, porous, and continuous three-dimensional network. This structure was characterized by disordered physical cross-linking and sponge-like micro-voids, potentially offering a high specific surface area. In contrast, SEPS-2 displayed a distinct morphology consisting of interwoven lamellar and coarse fibrillar structures, with a denser surface compared to SEPS-1, marked by significant wrinkling and interconnected ridges. Notably, SEPS-3 underwent a fundamental morphological transition, appearing as a highly compacted, irregular, and block-like aggregate with a remarkably smooth surface and minimal porosity. At 20,000× magnification, SEPS-3 revealed a granular texture with clearly defined, crystalline-like geometric contours, consistent with its high relative crystallinity. The distinct structural features-ranging from a porous microscopic grid in SEPS-1 to a dense micro-block in SEPS-3-highlight the profound morphological heterogeneity inherent in the SEPS, which is strictly governed by the molecular weight distribution of the polysaccharide chains.

### 3.8. XPS Surface Profiling

**Figure 6:**
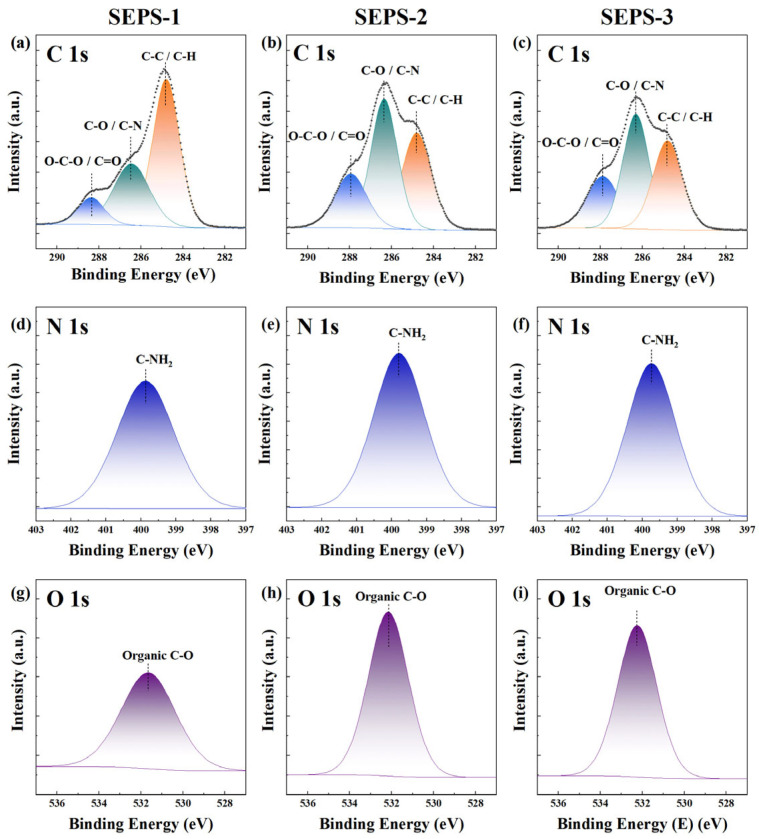
High-resolution C 1s, N 1s, and O 1s XPS spectra of different SEPS fractions. (**a**,**d**,**g**) SEPS-1; (**b**,**e**,**h**) SEPS-2; and (**c**,**f**,**i**) SEPS-3.

The surface elemental compositions and bonding states of SEPS fractions were analyzed via X-ray photoelectron spectroscopy (XPS) (Figure 6, Table 3). The high-resolution C 1s spectra were resolved into three distinct chemical environments: C-C/C-H (284.8 eV), C-O/C-N (286.5 eV), and O-C-O/C=O (288.5 eV). SEPS-1 exhibited a significantly higher atomic percentage of C-C/C-H (37.17%) compared to the other fractions, potentially reflecting a higher proportion of aliphatic or hydrophobic chain segments. SEPS-2 showed the maximum proportion of C-O/C-N (27.31%), while SEPS-3 displayed a marked increase in the O-C-O/C=O signal (14.39%), consistent with its enriched glycan backbone and uronic acid content. The N1s spectra for all fractions revealed a single symmetric peak near 400.0 eV, corresponding to organic nitrogen (C-NH_2_) in proteinaceous moieties, with atomic percentages ranging narrowly between 5.13% and 5.69%. The O1s peaks remained stable, with atomic proportions increasing slightly with molecular weight (from 29.58% in SEPS-1 to 31.47% in SEPS-3). The S2p signals remained negligible (0.23% −0.27%) across all samples, with a binding energy of 168.6 eV consistent with sulfate groups (−SO_4_^2−^), further confirming the low-sulfated nature of these SEPS.

### 3.9. Rheological Properties

**Figure 7:**
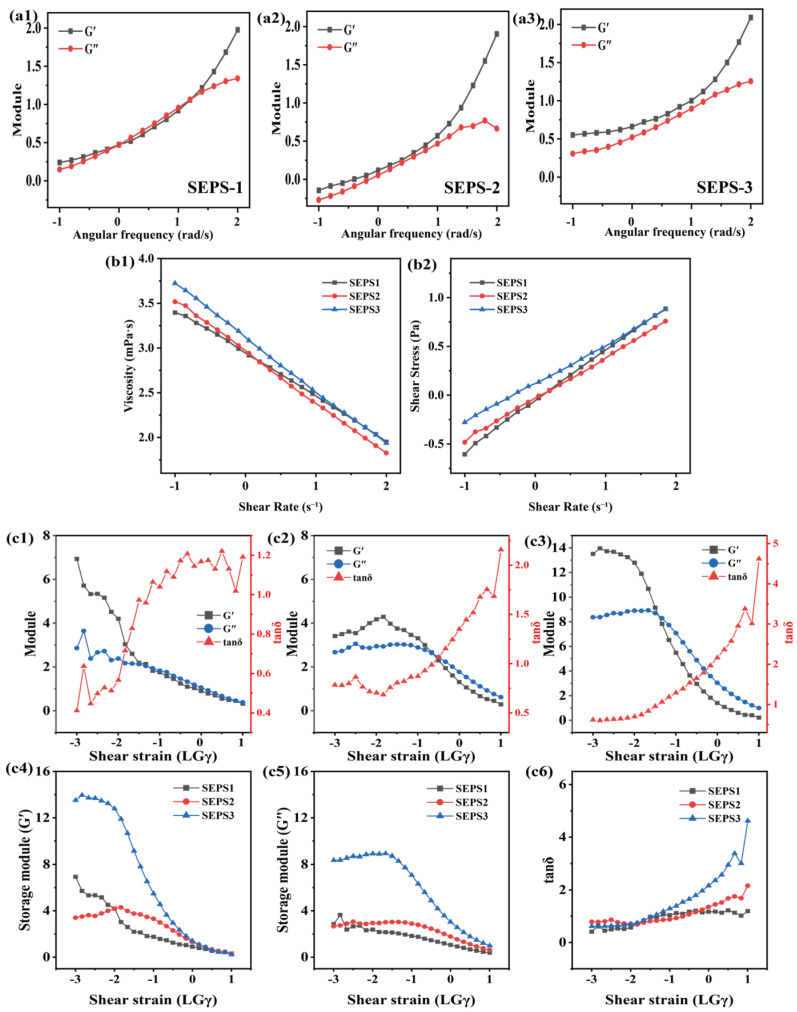
Rheological characterization of SEPS fractions. (**a1**–**a3**) Frequency sweeps of G′ and G″ vs. angular frequency. (**b1**,**b2**) Steady shear flow: (**b1**) apparent viscosity and (**b2**) shear stress vs. shear rate. (**c1**–**c6**) Amplitude sweeps: (**c1**–**c3**) G′, G″, and tanδ vs. shear strain; (**c4**–**c6**) comparison of viscoelastic parameters between fractions.

The viscoelastic behaviors and flow characteristics of the three SEPS fractions were systematically evaluated through amplitude sweeps, frequency sweeps, and steady shear tests (Figure 7). Amplitude sweeps (Figure 7c1–c6) defined the linear viscoelastic (LVE) region. For all samples, the storage modulus (G′) remained stable at low shear strains (*γ*< 1%, *log_10_γ* < −2), indicating a robust internal network. Notably, SEPS-3 exhibited the highest G′ (~14 Pa) and loss modulus (G″) within the LVE region (Figure 7c3–c5), significantly outperforming SEPS-1 and SEPS-2, which correlates with its higher molecular weight and enhanced intermolecular entanglements.

As the strain exceeded the critical yield point, a sharp decline in both moduli was observed, accompanied by a rapid increase in the loss tangent (*tanδ* > 1) for all fractions (Figure 7c1–c3,c6), signaling a structural transition from a solid-like to a liquid-like state. Frequency sweeps (Figure 7a1–a3) further elucidated the structural stability of the SEPS. Within the angular frequency range of 0.1–100 rad/s, G′ and G″ for all fractions increased with frequency. Notably, in SEPS-3 (Figure 7a3), G′ remained consistently higher than G″ across the tested range, a typical characteristic of a “weak-gel” structure. In contrast, SEPS-1 and SEPS-2 exhibited a crossover of G′ and G″ at higher frequencies (Figure 7a1,a2), suggesting a more fluid-like behavior dominated by molecular entanglements. Furthermore, steady shear tests (Figure 7b1,b2) demonstrated that the apparent viscosity (*η*) of all fractions decreased linearly with the increasing shear rate (*γ̇*) on a logarithmic scale (Figure 7b1). This pronounced shear-thinning behavior confirms the pseudoplastic nature of the SEPS solutions. SEPS-3 consistently maintained the highest viscosity and shear stress (*τ*) throughout the shear rate range (Figure 7b2), followed by SEPS-1 and SEPS-2.

### 3.10. Antioxidant Activity

**Figure 8:**
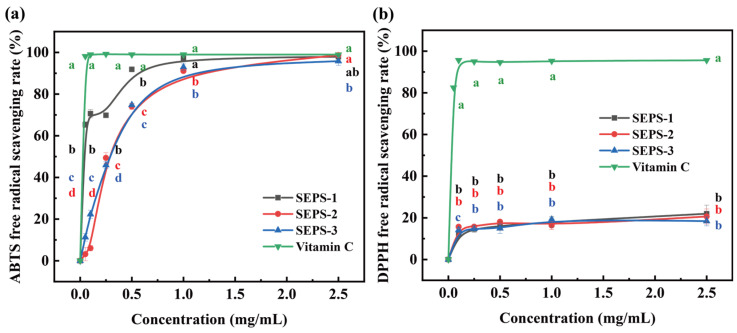
Comparisons of DPPH (**a**) and ABTS (**b**) radical scavenging rates of SEPS fractions. Different lowercase letters (a–d) above the bars indicate significant differences among the three fractions within the same category (*p* < 0.05). Error bars represent standard deviation (SD) from three independent replicates.

The antioxidant activities of the SEPS fractions were quantitatively evaluated via ABTS and DPPH radical scavenging assays, with Vitamin C serving as the positive control (Figure 8). All fractions exhibited concentration-dependent scavenging effects; however, their performance differed dramatically between the two radical systems and when compared to Vitamin C.

In the ABTS assay (Figure 8a), Vitamin C displayed the near-instantaneous scavenging rate of approximately 100% even at the lowest concentration tested (0.05 mg/mL). Among the SEPS fractions, SEPS-1 exhibited a marked advantage in both scavenging efficiency and potency. SEPS-1 reached a clearance rate exceeding 60% at 0.05 mg/mL and approached a plateau of nearly 100% at concentrations above 1.0 mg/mL, demonstrating efficacy comparable to Vitamin C in the high-concentration region. In contrast, SEPS-2 and SEPS-3 showed closely overlapping curves throughout the concentration range, characterized by a more gradual, linear increase. Their scavenging rates reached 74.02–74.80% at 0.5 mg/mL and 95.80–98.69% at the highest concentration (2.5 mg/mL). In the DPPH assay (Figure 8b), the scavenging capacities of all SEPS fractions were significantly weaker than those observed for ABTS. While Vitamin C consistently maintained a stable scavenging rate near 95% above 0.1 mg/mL, the SEPS fractions yielded low clearance values across all concentrations. Following a sharp initial increase to approximately 15% below 0.1 mg/mL, the curves for SEPS-1, SEPS-2, and SEPS-3 plateaued and remained clustered within the narrow range of 15% to 20% even at 2.5 mg/mL, indicating a limited DPPH radical scavenging capacity for these *L. platensis* SEPS under the tested conditions.

This pronounced discrepancy between the two radical systems is primarily attributed to the differing reaction media and underlying chemical mechanisms. DPPH assays are typically conducted in alcoholic media (e.g., ethanol or methanol), where polysaccharides exhibit poor solubility and a high tendency for self-aggregation. Such physical aggregation induces severe steric hindrance, which prevents the DPPH radicals from accessing the internal antioxidant sites within the glycan chains [41]. Conversely, the ABTS assay is performed in an aqueous system where the SEPS are fully hydrated and extended, facilitating more efficient interaction between the radicals and the antioxidant functional groups [42,43].

## 4. Discussion

### 4.1. Structural Partitioning and Elemental Fingerprinting

The elemental stoichiometry of SEPS fractions reveals a distinct molecular weight (MW)-dependent partitioning of proteinaceous impurities. In this study, the C/N ratio-a high-fidelity proxy for glycan purity-reached its maximum in SEPS-3, signifying an exceptionally intact polysaccharide backbone with minimal peptidic residues. Conversely, the depressed C/N ratio in SEPS-1, coupled with the high surface nitrogen density (N1s signals) observed in XPS analysis, supports a structural model of complex nitrogenous glycoconjugates. This is consistent with the findings of Páez-Watson et al. [44], who demonstrated that microbial EPS are frequently composed of glycoconjugates that exceed simple genomic predictions.

Furthermore, the hydrogen enrichment relative to other microbial EPS [45]—is likely a structural hallmark of *L. platensis* EPS, stemming from the high abundance of methyl-containing deoxy-sugars (Rha and Fuc). While the elevated C:H ratio in SEPS-1 implies a more condensed or unsaturated carbon framework, the ratios for SEPS-2 and SEPS-3 strictly align with the theoretical stoichiometry of standard carbohydrates [(CH_2_O)_n_] [46]. Notably, the negligible sulfur content (<0.3%) identifies these as non-sulfated polysaccharides, suggesting that their bioactivity is governed by the spatial accessibility of hydroxyl (-OH) and carboxyl (-COOH) groups rather than sulfation patterns.

### 4.2. Functional Stratification: From Biological Shield to Food Matrix Engineering

The biochemical profiles suggest that SEPS undergo rigorous functional stratification based on their MW and composition, with distinct implications for food applications (Table 4).

Enriched with deoxy-sugars, SEPS-1 (<30 kDa) possesses a loose, porous network and exhibits rapid ABTS scavenging activity (>60% at 0.05 mg/mL, ~100% at >1.0 mg/mL). Notably, this fast-acting antioxidant function is absent in commercial polysaccharides such as xanthan gum, pectin, and alginate. Therefore, SEPS-1 holds considerable promise for applications in functional beverages, natural antioxidant systems, and fast-dissolving dietary supplements [47,48]. Besides, ABTS assay may better reflect the antioxidant contents in SEPS fractions, which can be attributed to the polysaccharides being water-soluble but insoluble in ethanol.

SEPS-2 (30–100 kDa) identifies as an amphiphilic glycoprotein complex (17.40% protein). Although XRD profiles indicate high crystallinity (70.58%)—likely due to protein-templated local ordering—the intense mass loss in TGA suggests that the protein-polysaccharide interface is thermally sensitive. Structurally, the pronounced Congo red shift (λ_max_ = 514 nm) and dense hydrogen-bonding network (FT-IR) enhance interfacial adsorption. Collectively, these properties confer natural emulsifying and interface-stabilizing functionality [49]. In contrast, xanthan gum and alginate exhibit weak emulsifying capacity, highlighting the unique advantage of SEPS-2. Consequently, it is well suited for salad dressings, plant-based milk alternatives, whipped cream, and high-temperature-processed emulsion systems.

SEPS-3 (>100 kDa) has the highest C/N ratio (10.015), indicating a high-purity glucan backbone (28.86% glucose). Moreover, its ultra-high molecular weight enables extensive chain entanglement and a weak-gel structure (G′ consistently > G″), complemented by a dense, non-porous, block-like morphology. Functionally analogous to xanthan gum in shear-thinning behavior, SEPS-3 nevertheless differs fundamentally in its entanglement-based gelation mechanism [47]. While functionally analogous to xanthan gum in shear-thinning behavior, SEPS-3 provides superior film-forming density, making it ideal for edible preservative coatings that hinder moisture and oxygen transport, thereby extending the shelf-life of perishable produce.

### 4.3. Synergistic Implications for Sustainable Food Production

Spectroscopic and thermodynamic analyses provide macroscopic validation of this structural heterogeneity. FT-IR spectra revealed that SEPS-2 possesses a denser hydrogen-bonding network, correlating with its high rhamnose and fucose content, which likely alters the spatial arrangement to expose more hydroxyl sites. XRD profiles further confirm that SEPS-2 achieves the highest crystallinity (70.58%), likely due to protein-induced local ordered orientation, which explains the sudden, intense mass loss observed in thermal analysis (TGA). While SEPS-3 exhibits slightly lower crystallinity due to topological constraints from its massive supramolecular weight (>100 kDa), its dense, non-porous bulk morphology observed via SEM confirms its role as a structural architect. The elevated baseline in the UV-Vis visible spectrum for SEPS-3 is a manifestation of Mie scattering rather than chromophore density; this optical evidence confirms that SEPS-3 constructs a physical barrier capable of intercepting light energy and hindering the photosynthetic efficiency of the culture. The functional difference of SEPS fractions allows *L. platensis* to survive in complex environments: low-MW fractions sacrifice structural stability for rapid chemical protection, while high-MW fractions construct a durable physical shield. This ecological insight provides a theoretical foundation for targeted metabolic modulation in industrial biorefineries.

For large-scale biomass production, the accumulation of high-MW SEPS-3 may hinder photosynthetic efficiency via light scattering; however, these same fractions represent the most valuable ingredients for food texture engineering. Therefore, a dual-purpose strategy is proposed: by implementing strategic enzymatic degradation or precision filtration of the 30–100 kDa (SEPS-2) and > 100 kDa (SEPS-3) fractions, producers can simultaneously maintain culture vitality and harvest high-purity, bio-active polysaccharides. This approach transforms SEPS from a metabolic byproduct into a high-performance functional food platform, bridging the gap between algal biotechnology and precision food science. Despite the high application potential of EPS, its industrial-scale adoption is constrained by high synthesis costs and low natural fermentation yields [50,51]. Moving forward, rigorous attention must be paid to ensuring inter-batch consistency of SEPS to meet strict industrial standardization requirements. Simultaneously, contemporary research is increasingly harnessing metabolic and genetic engineering strategies to directionally induce the production of specific SEPS fractions with desired molecular weights and functional attributes, thereby optimizing the high-value utilization. These biotechnological advancements are expected to improve EPS quality and cost-efficiency, facilitating its transition from laboratory research to widespread commercial application.

## 5. Conclusions

This study establishes a molecular weight (MW)-driven blueprint for functional diversification of *Limnospira platensis* extracellular polysaccharides (SEPS), positioning them as versatile candidates from non-food biomass for the food industry. The low-MW SEPS-1, with its high antioxidant activity, emerges as a natural source for food preservatives or health-promoting supplements. The mid-MW SEPS-2, an amphiphilic glycoprotein, holds promise as a bio-emulsifier or stabilizer in complex food matrices. The high-MW SEPS-3, forming robust physical networks, showcases potential as a biodegradable structuring agent or viscosity modifier. Importantly, identifying the 30–100 kDa fraction as a dual-functional entity-both a potential inhibitor in cultivation and a source of unique interfacial properties-provides a critical lever for optimization. For sustainable *L. platensis* production, targeted management of this fraction can enhance medium recyclability. For food application, its isolation offers a distinct functional ingredient. Future research should focus on in vitro and in vivo validation of these hypothesized food functionalities (e.g., emulsification stability, prebiotic activity, glycemic response modulation), toxicological safety assessments, and integration of SEPS fractions into model food systems to evaluate their techno-functional performance and consumer acceptance. This work underscores the potential of leveraging microalgal by-products from circular cultivation processes to create a new portfolio of sustainable, functional food ingredients.

## Figures and Tables

**Table 1:** Elemental content ratio of SEPS fractions

Ratio	C/H	C/N	H/N	S
SEPS-1	5.738 ± 0.134 ^a^	9.207 ± 0.614 ^b^	1.607 ± 0.145 ^b^	<0.3%
SEPS-2	4.978 ± 0.143 ^b^	9.344 ± 0.069 ^a,b^	1.878 ± 0.068 ^a^	<0.3%
SEPS-3	5.151 ± 0.108 ^b^	10.015 ± 0.009 ^a^	1.945 ± 0.043 ^a^	<0.3%

Data are expressed as mean ± SD (*n* = 3). Different superscript letters (^a^,^b^) within the same column indicate significant differences among the three SEPS fractions (*p* < 0.05).

**Table 2:** Crystallinity of SEPS fractions.

Fraction	SEPS-1	SEPS-2	SEPS-3
Relative crystallinity %	68.82%	70.58%	69.96%

**Table 3:** Surface elemental composition and C1s peak deconvolution of SEPS fractions.

Fractions			SEPS-1	SEPS-2	SEPS-3
Response Value	C-C/C-H		48,260.53	31,657.38	29,102.51
	C-O/C-N		20,144.93	42,405.55	37,560.03
	O-C-O/C=O		8795.85	17,719.68	17,033.65
Atomic Percentage (%)	S2p	168.6 eV	0.27%	0.23%	0.24%
	C1s	284.8 eV	37.17%	22.58%	22.60%
	C1s Scan A	286.5 eV	20.72%	27.31%	25.73%
	C1s Scan B	288.5 eV	6.57%	13.58%	14.39%
	N1s	399.9 eV	5.69%	5.13%	5.56%
	O1s	532.3 eV	29.58%	31.18%	31.47%

**Table 4:** Functional profile and food industry applications of SEPS fractions.

Fraction	Key Chemical Feature	Functional Role	Potential Food Application
SEPS-1	Low MW (<30 kDa), Deoxy-sugars	Fast-acting Antioxidant	Functional beverages, dietary supplements
SEPS-2	Amphiphilic Glycoprotein, 70% Crystallinity	Natural Emulsifier	Salad dressings, plant-based milk, whipped cream
SEPS-3	High MW (>100 kDa), Weak-gel structure	Film-forming & Thickening	Edible coatings for fruit preservation, viscosity modifiers

## Data Availability

The data presented in this study are available on request from the corresponding author. The data are not publicly available due to privacy.

## Abbreviations

The following abbreviations are used in this manuscript:

EPS	Exopolysaccharides
SEPS	*Spirulina* Exopolysaccharides
*L. platensis*	*Limnospira platensis*
OMs	Organic Matters
MW	Molecular weight
UV-Vis	Ultraviolet-Visible Spectroscopy
FT-IR	Fourier Transform Infrared Spectroscopy
TGA	Thermal analysis
DTG	Derivative Thermogravimetry
SEM	Scanning Electron Microscope
DPPH	2,2-Diphenyl-1-picrylhydrazyl
ABTS	2,2′-Azinobis-(3-ethylbenzthiazoline-6-sulphonate)
SR	Scavenging rates
XRD	X-ray diffraction
XPS	X-ray photoelectron spectroscopy
LVE	Linear viscoelastic region
